# Functional traits driving invasion risk and potential distribution of alien plants in oasis agroecosystems

**DOI:** 10.3389/fpls.2025.1590709

**Published:** 2025-05-19

**Authors:** Shengtianzi Dong, Tiantian Qin, Zhifang Xue, Wenchao Guo, Hanyue Wang, Hongbin Li

**Affiliations:** ^1^ The College of Life Sciences, Shihezi University, Shihezi, China; ^2^ The Key Laboratory of Oasis Town and Mountain-basin System Ecology, Xinjiang Production and Construction Corps, Shihezi, China; ^3^ Xinjiang Key Laboratory of Agricultural Biosafety, Urumqi, Xinjiang, China

**Keywords:** biological invasion, functional traits, invasion risk, oasis agriculture, potential distribution

## Abstract

Alien invasive plants pose a significant threat to global agricultural production, with functional traits playing a critical role in their spread and establishment processes. However, relevant research is scarce in oasis agroecosystems, which are more sensitive to global change. We studied oasis agroecosystems in Xinjiang, China, to explore the relationship between alien plant functional traits and invasion risk. A total of 611 sites comprising 9,165 plots were surveyed, covering an area of 22,474.73 hectares. Field surveys recorded species, density, and cover of alien plants, measuring traits related to growth, reproduction, and dispersal. Invasion risk was classified into four levels based on importance values. Random forest and eXtreme Gradient Boosting (XGBoost) modeling analyzed the relationship between functional traits and invasion risk, while MaxEnt modeling predicted potential distributions. We identified 62 alien plant species from 18 families and 44 genera, with Asteraceae and Amaranthaceae being the most represented families. High-risk invasive plants shared certain functional traits—specifically, high specific leaf area (SLA) and larger seed mass—which significantly enhance their invasion potential in oasis agroecosystems. The combination of these traits correlates with increased invasion risk. By incorporating SLA into the weighting of high-risk species distributions, we predicted potential distribution areas with an AUC value of 0.981. Our study identifies key functional traits enabling alien plant invasions in oasis agriculture, enhancing understanding of invasion mechanisms. Findings provide a foundation for predicting potential invasive species and developing management strategies to mitigate impacts on agricultural productivity and ecosystem services.

## 1 Introduction

Biological invasions constitute a global threat to biodiversity and ecosystem services, exerting particularly severe impacts on agricultural systems through crop suppression, habitat alteration, and economic burdens (Funk et al., 2014; Liu Y. et al., 2017; Campbell et al., 2023; Chu et al., 2024). In arid regions worldwide, oasis agriculture emerges as a vital yet vulnerable food production system. Characterized by intensive irrigation on desert peripheries, these agroecosystems paradoxically combine concentrated resources, frequent human disturbances, and sharp environmental gradients—conditions that, despite the surrounding aridity, facilitate alien plant invasions (Milton and Dean, 2010; Oduor et al., 2016; Liu Y. et al., 2017; Tesfay et al., 2023). Such invasions jeopardize not only crop yields but also the ecological balance of these water-limited lifelines (Campbell et al., 2023; Lenzner et al., 2024).

The invasion dynamics in oasis agriculture can be framed within ecological niche theory and resource fluctuation hypothesis (Shea and Chesson, 2002; Macdougall et al., 2009; Mallon et al., 2015). While traditional invasion theories suggest that environmental stability promotes resistance to invasion, the distinctive ecological conditions of oasis agroecosystems—specifically the juxtaposition of resource-rich oasis zones against resource-poor desert peripheries—create unique invasion opportunities through niche differentiation and resource pulses (Har-Edom and Sternberg, 2010; Davis et al., 2017; Staubus et al., 2019). While aridity generally restricts plant colonization (Har-Edom and Sternberg, 2010; Davis et al., 2017; Staubus et al., 2019), the resource-rich mosaic of irrigated fields, canals, and managed landscapes creates “invasion hot-spots” where alien species bypass typical environmental filters (Milton and Dean, 2010; Yuan et al., 2019; Fan et al., 2020; Sun et al., 2021). This intermediately disturbed landscape facilitates invasions through what we conceptualize as “functional trait filtering”—a process whereby specific trait combinations enable aliens to exploit the resources provided by oasis environments while withstanding the stresses of the surrounding arid matrix (Drenovsky et al., 2012; El-Barougy et al., 2020). This dichotomy highlights the need for invasion ecology frameworks specifically tailored to oasis environments, particularly through the lens of functional trait analysis - a critical tool for predicting invasion success yet understudied in arid agroecosystems (Valencia et al., 2015; Díaz et al., 2016; De Oliveira et al., 2020).

Plant functional traits mediating resource acquisition and stress tolerance (Soudzilovskaia et al., 2013; Díaz et al., 2016; Hulme and Bernard-Verdier, 2018) may determine invasion success in oasis settings. Rapid growth rates, efficient water-use strategies, and disturbance tolerance have been implicated in agricultural invasions globally (Godoy et al., 2012; Nunez-Mir et al., 2019; Montagnani et al., 2022), but their relative importance under oasis conditions remains unclear (Valencia et al., 2015; De Oliveira et al., 2020). Based on trait-mediated invasion phenomena, we hypothesize that successful invaders in oasis agroecosystems likely possess similar functional trait combinations that optimize exploitation of resource-rich habitats while maintaining tolerance to the harsh climatic extremes characteristic of desert margins.

Our study focuses on oasis agroecosystems regions in Xinjiang, China, aiming to explore the relationship between alien plant functional traits and invasiveness in these agroecosystems. Through field surveys and model analyses, we aim to: (1) Identify alien plant species in oasis agriculture and classify their invasion risk; (2) Determine which functional traits contribute to high invasion risk; and (3) Predict potential distributions of these alien plants based on key functional traits. This research enhances understanding of plant invasions in oasis agroecosystems and is crucial for predicting potential invasive species and implementing targeted management to protect agricultural productivity and ecosystem services.

## 2 Materials and methods

### 2.1 Study area overview

**Figure 1:**
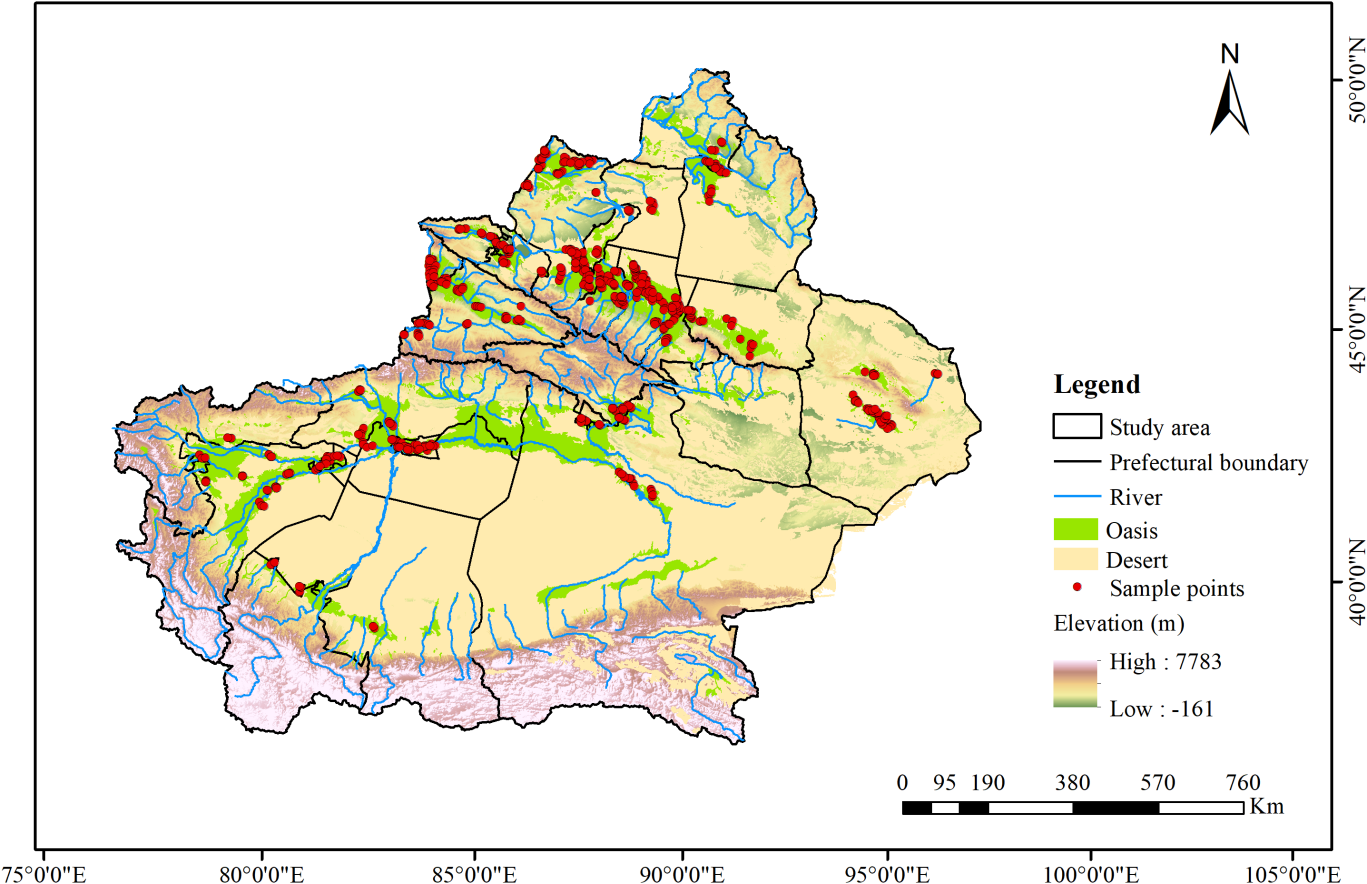
Distribution map of study area points. A total of 611 locations were selected across all oasis regions for data analysis.

The study area is the Xinjiang, China, located between 73°40’ - 96°18’ E and 34°25’ - 48°10’ N. This region is characterized by a typical continental arid climate and ecological fragility, with interspersed oases and deserts. It features low precipitation, high evaporation, and significant diurnal temperature variation. The average annual precipitation is about 155 mm, sometimes dropping below 10 mm, while evaporation can be dozens of times greater (Zhang et al., 2022). This study conducted surveys across the oases and the transitional zones between oases and deserts, setting up 611 survey sites (**Figure 1**) to identify alien plant species present and classify their risk levels.

### 2.2 Sample survey

Considering the significant interannual variability in plant distribution in arid regions (Lázaro-Nogal et al., 2015; Yao et al., 2020) and potential seed bank effects (Ellner et al., 2016), a three-year field survey was conducted from 2022 to 2024 to gather comprehensive plant species data. Surveys were carried out from June to August each year, coinciding with the peak growth period for most plants in this arid region. We established 611 survey sites (**Figure 1**) across different land use types, including farmland, orchards, urban and industrial areas, transportation land, and water and hydraulic facilities.

In each survey site, we used a multi-sampling approach. Initially, we conducted a reconnaissance to identify areas with dense plant distribution for plot setup. We then randomly selected three plots of 20 m×20 m or equivalent size. Within each plot, five 1 m×1 m quadrats were randomly established. We recorded the plant species within each quadrat, along with their respective densities and coverages. For each species, we selected over ten individuals in their reproductive phase to measure plant height, and collected ten healthy, intact leaves to measure SLA in the lab (Liu Z. et al., 2017). Based on literature and phenological data from the first year, seeds from all plant species at each site were collected over the three years. Seeds of the same species from each site were mixed and brought to the lab for measuring the seed hundred-grain weight.

### 2.3 Study on the species and invasion risk levels of alien plants

For the surveyed plants, we determined the native origin of each species by consulting various sources, such as the Flora of Xinjiang and Web of Science, to assess whether they are alien to the region.

Once the alien plant species were identified, we categorized their invasion risk levels based on the importance value (**Supplementary Table S1**). The invasion risk of these alien plants was classified into four levels according to the importance value, ranked from high to low (Catford et al., 2012). Species in the top 25% are considered high risk, those between 25% and 50% as medium risk, between 50% and 75% as low risk, and those beyond 75% as no risk (Catford et al., 2012; Osawa et al., 2019). The importance value was calculated using the following Equations 1–4:


(1) Importance Value = (Relative Density + Relative Frequency + Relative Coverage) / 3

(2) Relative Density=(Number of individuals of a specific alien plant/Total number of plant individuals)×100%

(3) Relative Frequency=(Number of plots in which a specific alien plant appears/Total number of plots)×100%

(4) Relative Coverage =(Coverage of a specific alien plant/Total coverage of all plants)×100%

### 2.4 Relationship between functional traits of alien plants and invasiveness

To identify which functional traits enhance the invasiveness of alien plants in arid regions, we analyzed growth-related traits (Xiao et al., 2019), reproductive traits (Burns et al., 2011), and dispersal traits (Jacobs and Lesmeister, 2012). Growth-related traits include life forms, SLA, and plant height. Reproductive traits encompass mating systems, flowering duration, and pollination methods. Dispersal traits involve seed hundred-grain weight, seed type, and dispersal vectors (**Supplementary Table S2**). Some of these trait indicators are continuous variables measured during our survey, while others are categorical variables obtained from relevant literature.

### 2.5 Predicting potential distribution based on functional traits

Traditional models for predicting species’ potential distribution rely on the presence or absence of target species at survey sites, combined with factors such as climate (Ashcroft et al., 2017; Chevalier et al., 2021). However, for species that are under-sampled, rare, or highly cryptic, these predictions may be inaccurate or biased. Additionally, this approach typically considers only macro-environmental factors like climate and topography, while neglecting interactions among organisms, species’ physiological and ecological traits, and evolutionary history, failing to fully capture the species’ true environmental responses.

Trait-based prediction methods effectively address these limitations. Functional traits refer to characteristics of species in terms of morphology, physiology, and ecology that directly affect their interactions with the environment (Bertelsmeier, 2017). By analyzing these traits, prediction models can better understand the mechanisms by which species respond to environmental factors. We used the functional traits identified as enhancing the invasiveness of alien plants in arid regions to weight the distribution points of species, predicting the potential distribution of these alien plants in the study area.

### 2.6 Data analysis

#### 2.6.1 Relationship between functional traits of alien plants and invasiveness

To identify the functional traits that contribute to the invasion risk of alien plants in oasis agroecosystems, we employed two complementary machine learning approaches: Random Forest and Extreme Gradient Boosting (XGBoost) models in R software (version 4.4.2). Both models are well-suited for handling mixed data types (continuous and categorical variables), meeting the data processing requirements of this study. In our models, the dependent variable is invasion risk, categorized into no risk, low risk, medium risk, and high risk. The independent variables are all the functional traits identified earlier.

Model Construction: During data preprocessing, we converted the dependent variable and all categorical independent variables into factor types. For XGBoost analysis, categorical variables were further transformed using one-hot encoding to create a comprehensive feature matrix. The dataset was then split into a training set and a test set in a 70% to 30% ratio. Using the randomForest and xgboost packages in R, we built both models. To optimize model performance, we applied cross-validation to adjust model parameters. For the Random Forest model, we optimized the number of trees (ntree) and the number of variables tried at each split (mtry). For the XGBoost model, we optimized key hyperparameters including learning rate (eta), maximum tree depth (max_depth), minimum child weight, and sampling rates for observations and features.

Model Evaluation: We assessed performance of both models on the test set by calculating metrics such as Accuracy, Precision, Recall, and F1-score. Confusion matrices were generated to analyze each model’s predictive effectiveness for each risk level, allowing for direct comparison between the two approaches.

Model Validation and Robustness Analysis: To verify the robustness of both models, we conducted multiple repeated experiments, changing the random seed to test the consistency of model performance. We further optimized model parameters through cross-validation to ensure good generalization ability across different data subsets.

Variable Importance Analysis: To ensure comparability between the two models, we employed a model-agnostic approach to feature importance calculation using the DALEX framework. This allowed us to generate standardized importance metrics across both Random Forest and XGBoost models, providing a unified assessment of which functional traits most significantly contribute to invasion risk prediction. Variable importance plots were created to visually display and compare the ranking of trait importance between models, determining which traits consistently impact increased invasion risk across different modeling approaches.

Analysis of Key Functional Traits Across Invasion Risk Levels: To examine the relationship between the top two most important functional traits and invasion risk levels, we conducted one-way Analysis of Variance (ANOVA) tests. Prior to analysis, data were checked for normality using the Shapiro-Wilk test and for homogeneity of variance using Levene’s test. For cases where assumptions could not be met, we employed the non-parametric Kruskal-Wallis test as an alternative. For *post-hoc* comparisons, we used Tukey’s Honestly Significant Difference (HSD) test to identify significant differences between invasion risk categories (no risk, low risk, medium risk, and high risk). Statistical significance was established at p< 0.05.

#### 2.6.2 Trait-weighted potential distribution

To predict the potential distribution of alien plants with specific functional traits in the oasis agroecosystems of Xinjiang and identify key management areas, we employed a trait-based species distribution model. Data analysis was conducted using R software (version 4.4.2). We focused on key functional traits that enhance invasiveness, first using RLQ analysis to identify traits that best respond to environmental gradient changes, and further selected traits for potential distribution prediction. Then, the MaxEnt model was used for prediction.

Model Construction: During data preprocessing, we first normalized the functional trait data and calculated a composite TraitScore. This score was assigned as a weight to the presence points of the corresponding species. Climate raster data from the Xinjiang region were used as environmental variables.

We combined the species presence points with the weighted TraitScore data to generate a weighted presence dataset. To construct the model, we randomly generated background points within the study area, totaling ten times the number of presence points, to provide the necessary background environmental information for model training.

In terms of data division, the dataset was split into a training set and a test set in a 70% to 30% ratio, ensuring the reliability of model construction and evaluation. Using the maxnet package in R, we built a weighted MaxEnt model based on the training data. In this model, environmental variables served as independent variables, species presence as the dependent variable, and weight values emphasized the importance of different presence points to accurately reflect the impact of functional traits on species distribution.

Model Evaluation: The predictive performance of the model was assessed on the test set. The model was evaluated using the receiver operating characteristic (ROC) curve and the area under the curve (AUC) value. An AUC value closer to 1 indicates stronger predictive ability of the model.

## 3 Results

### 3.1 Identification of alien plant species and invasion risk classification based on importance value

**Figure 2:**
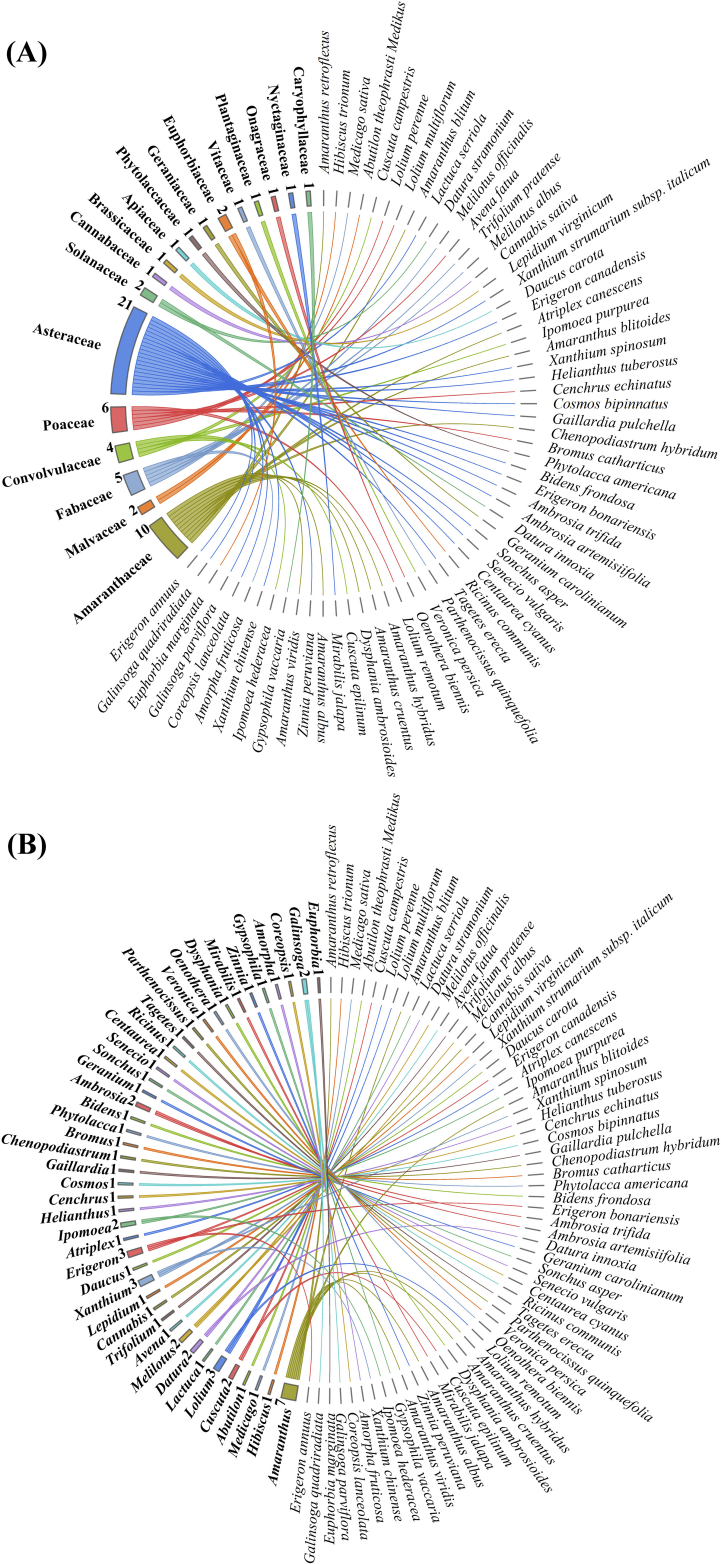
Chord diagram of the classification of 62 species of alien plants. **(A)** shows the classification of species at the family level, comprising 18 families; **(B)** illustrates the classification at the genus level, including 44 genera.

In the study area, a total of 62 alien plant species were identified, belonging to 18 families and 44 genera (**Figure 2**). At the family level, Asteraceae was the predominant family, with 21 species, accounting for 33.9% of all alien plants. This was followed by Amaranthaceae with 10 species, representing 16.1%. There were 10 monotypic families. At the genus level, *Amaranthus* was the dominant genus, with 7 species, making up 11.3% of all alien plants. There were 34 monotypic genera, accounting for 54.8% of all alien invasions. Overall, the distribution of alien plants across families and genera showed a clear concentration, with a few families and genera containing most invasive species, while many had only one species.

A total of 611 sites comprising 9,165 plots were surveyed, covering an area of 22,474.73 hectares. By calculating the relative density, relative frequency, and relative coverage of each species, the importance values were obtained. These values were then ranked from high to low and classified into four invasion risk levels (**Table 1**): severe risk (15 species), moderate risk (15 species), low risk (16 species), and no risk (16 species). Among the severely invasive alien plants, most were from the Asteraceae family (5 species), followed by the Fabaceae family (3 species).

**Table 1:** The invasion risk level classification of alien plants based on the importance value.

Class	Average Relative Density	Average Relative Frequency	Average Relative Cover	Average Importance Value
High	3.094	16.231	9.293	9.539**
Medium	0.350	1.830	1.408	1.196*
Low	0.022	0.423	0.407	0.284
No	0.004	0.098	0.091	0.064

*P<0.05, **P<0.01

Based on the importance values from highest to lowest, divide into four risk levels using the mean quartile method. For each level, calculate the average relative density, relative frequency, and relative coverage of the species. These are then weighted to obtain the average importance value.

### 3.2 Relationship between functional traits of alien plants and invasiveness

**Figure 3:**
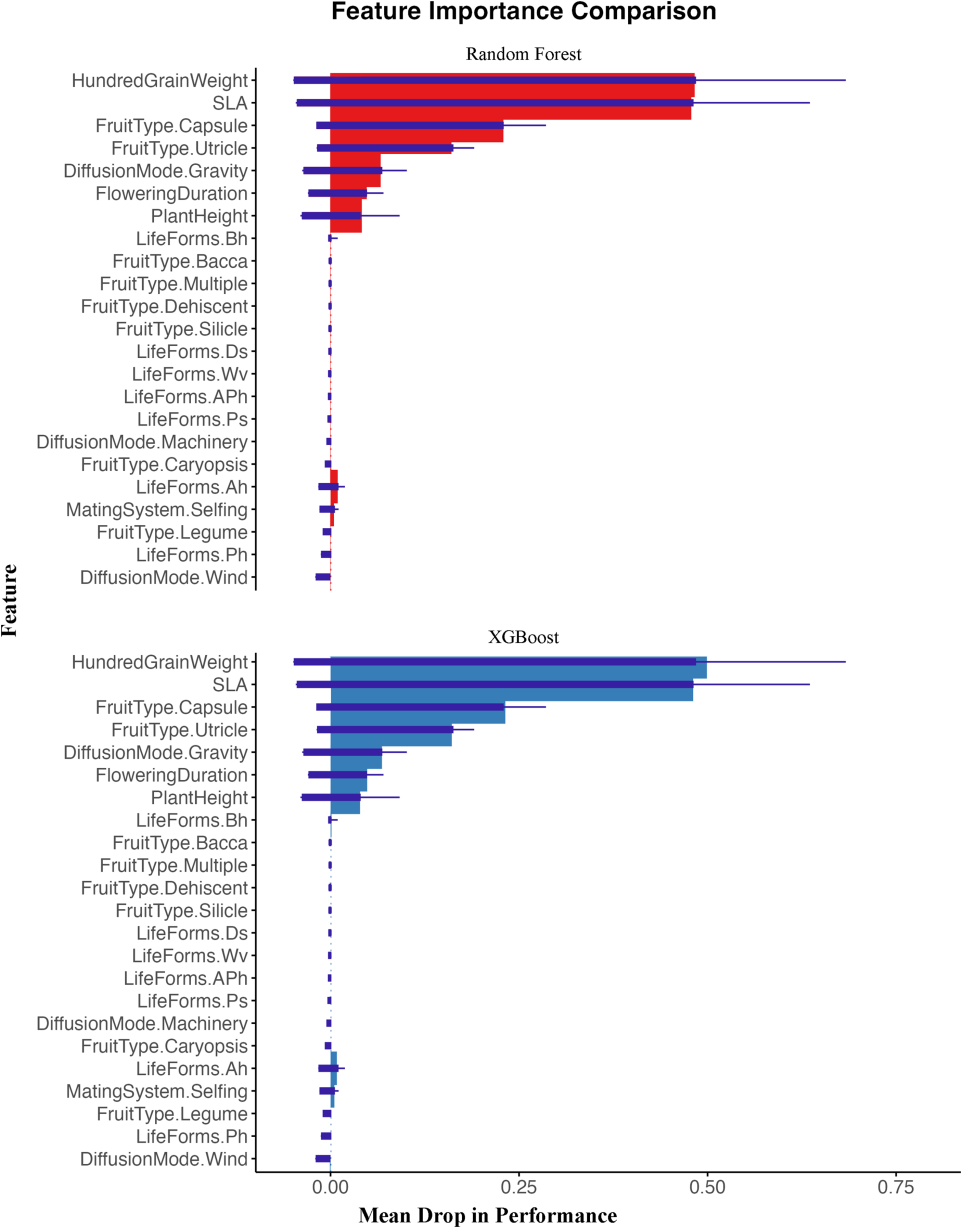
The importance of plant functional traits was represented by the Mean Drop in Performance using both random forest and XGBoost models. The figure is divided into two parts: the upper section (red) shows results from the Random Forest model, while the lower section (blue) represents the XGBoost model results. The length of each bar indicates the relative importance of features. The horizontal axis represents “Mean Drop in Performance,” which measures feature importance: the “0” point represents the baseline, indicating features with no effect on model prediction ability; positive values (right of “0”) indicate features that positively contribute to model predictions, with larger values signifying greater importance as removing these features causes more significant performance decline; negative values (left of “0”) would indicate that removing the feature actually improves model performance, suggesting the feature may introduce noise. The results indicate that Hundred-grain weight and SLA are the most important traits for predicting invasion risk across both models, followed by Fruit Type (Capsule), Fruit Type (Utricle), and Diffusion Mode (Gravity). See **Supplementary Table S2** for feature abbreviations.

**Figure 4:**
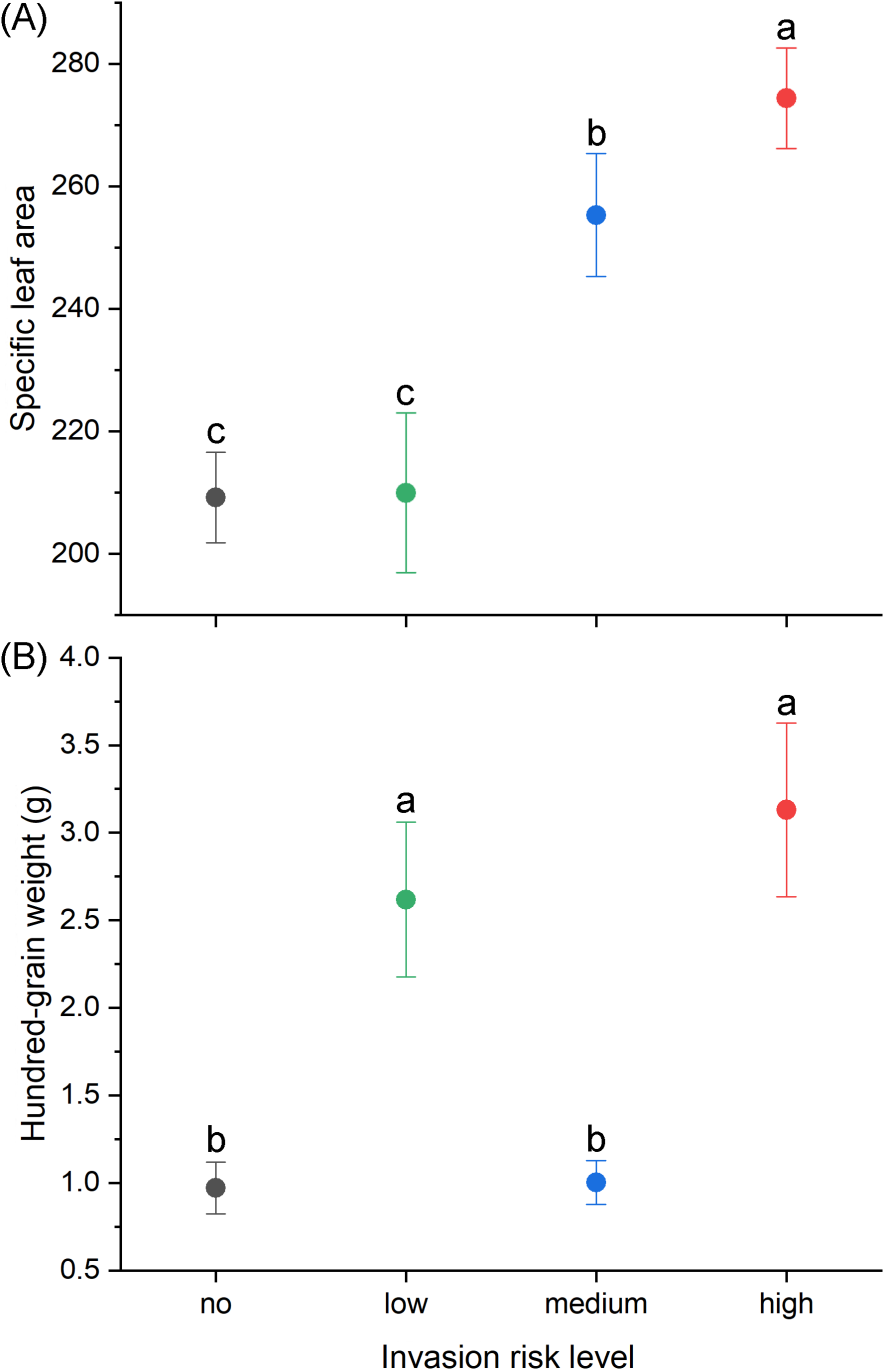
Relationship between invasion risk level and **(A)** specific leaf area and **(B)** hundred-grain weight. Different letters indicate significant differences among treatment groups at *P*< 0.05.

In this study, we used both random forest and XGBoost models to analyze the relationship between invasion risk and functional traits of alien plants in oasis agroecosystems. Both models produced consistent results, showing that SLA and seed hundred-grain weight ranked highest in importance metrics (**Figure 3**), indicating they are the most critical factors contributing to invasion risk. As invasion risk levels increased, SLA significantly increased, and high-risk alien plants had the highest average seed hundred-grain weight (**Figure 4**). Additionally, both models identified several other important traits that facilitate invasion, including Fruit Type (Capsule), Fruit Type (Utricle), and Diffusion Mode (Gravity).

The model evaluation demonstrated excellent performance for both approaches, confirming the reliability of these results. For the random forest model, the overall accuracy on the test set was 99.73%, with a Kappa coefficient of 0.9964, indicating a high consistency between model predictions and actual classifications (**Table 2**). Similarly, the XGBoost model achieved an accuracy of 99.72% with a Kappa coefficient of 0.9963 (**Table 3**). Precision, recall, and F1-score for each invasion risk category exceeded 98% in both models, showing balanced and accurate prediction performance across different risk levels. The consistency between these two different machine learning approaches (random forest and XGBoost) provides strong evidence that the identified functional traits are indeed the key factors determining invasion risk in oasis agroecosystems, with credible results.

**Table 2:** Performance of the random forest model on the test set.

Class	Precision	Recall	F1-Score
High	0.9989	0.9889	0.9944
Medium	0.9948	0.9894	1.0000
Low	1.0000	1.0000	1.0000
No	0.9897	1.0000	0.9948

Accuracy=0.9973, Kappa=0.9964. The higher the Precision, Recall, and F1_Score, the better the model’s predictive performance. Accuracy is the ratio of correctly predicted instances (both true positives and true negatives) to the total number of instances. It measures the overall correctness of the model. Kappa is a statistic that measures the agreement between the predicted and actual classifications, adjusted for the agreement that could happen by chance. Precision is the ratio of true positive predictions to the total number of positive predictions (both true positives and false positives). It indicates how many of the predicted positive instances were actually correct. Recall is the ratio of true positive predictions to the actual number of positive instances. It measures the model’s ability to correctly identify all relevant instances. The F1 Score is the harmonic mean of Precision and Recall. It provides a balance between the two metrics and is especially useful when the class distribution is imbalanced.

**Table 3:** Performance of the XGBoost model on the test set.

Class	Precision	Recall	F1-Score
High	1.0000	0.9872	0.9935
Medium	0.9890	1.0000	1.0000
Low	1.0000	1.0000	0.9944
No	1.0000	1.0000	1.0000

Accuracy=99.72%, Kappa=0.9963. The higher the Precision, Recall, and F1_Score, the better the model’s predictive performance.

### 3.3 Trait-weighted potential distribution prediction

**Figure 5:**
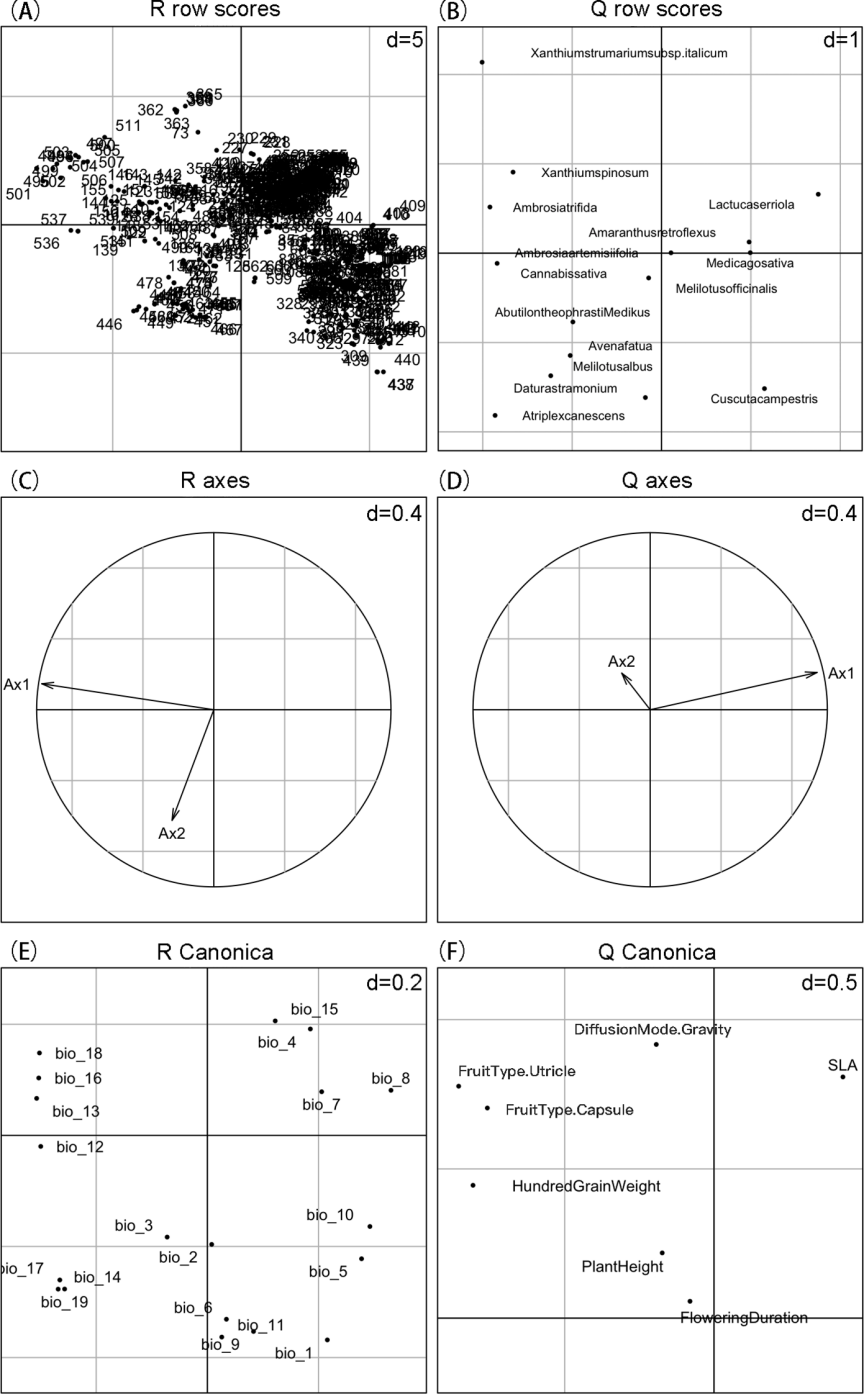
RLQ analysis results revealing the relationships among environmental variables, species functional traits, and species distribution. **(A, B)** represent the distribution of scores for each sampling site and species after principal component analysis (PCA) of the environmental variables (R table) and functional traits (Q table), respectively. **(C, D)** illustrate the correlations among variables. **(E)** shows the canonical scores of environmental variables in the RLQ analysis. **(F)** displays the canonical scores of functional traits in the RLQ analysis; SLA is positively correlated with the main environmental gradient, indicating a positive response of species to the environment.

Through RLQ analysis, we found that SLA is located in the first quadrant of the Q-score plot (**Figure 5**). This indicates a positive correlation between SLA and the main environmental gradient (first axis), allowing it to actively respond to climate gradient changes. Therefore, SLA can be considered a key functional trait for predicting species potential distribution based on climate factors. In contrast, hundred-grain weight, plant height, and flowering duration are positioned differently on the score plot, showing weaker or different correlations with the main environmental gradient. Given the close association between SLA and climate factors, we focused on SLA for potential distribution prediction to more accurately reflect species’ responses and adaptations to climate gradients.

**Figure 6:**
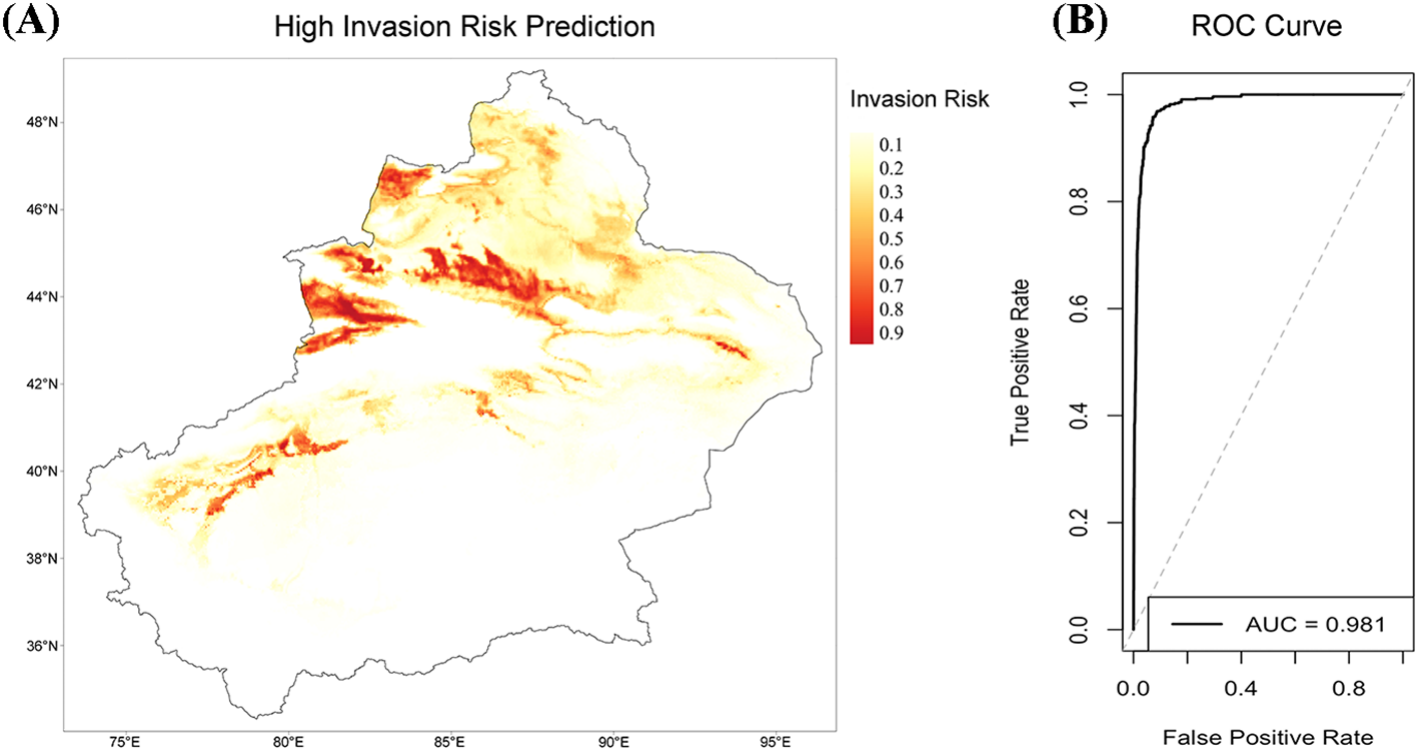
Species potential distribution risk prediction map weighted by SLA and the ROC curve of the MaxEnt model. **(A)** Potential distribution risk prediction map of 15 species weighted by normalized SLA values. The color gradient from light to dark indicates the variation in species distribution risk from low to high. Darker areas represent regions with higher suitability or distribution probability for the species. **(B)** Receiver Operating Characteristic (ROC) curve of the MaxEnt model, with an AUC = 0.981. The curve illustrates the true positive rate and false positive rate of the model under different classification thresholds. The area under the curve (AUC) being close to 1 indicates that the model has excellent discriminatory ability and high predictive accuracy.

In the potential distribution prediction study, we used normalized SLA values to weight the presence points of 15 high-risk invasive species, aiming to assess the impact of SLA on species distribution through the MaxEnt model (**Figure 6**). The weighted MaxEnt model demonstrated good predictive performance, with an AUC of 0.981 (**Figure 6**), indicating high discriminative ability, effectively distinguishing suitable and unsuitable habitats for the species.

## 4 Discussion

In recent years, plant invasion has significantly increased in arid regions, including oasis agricultural areas, challenging the traditional ecological view that arid environments naturally resist alien plants due to resource scarcity and harsh conditions (Har-Edom and Sternberg, 2010; Staubus et al., 2019). Our research directly addresses this paradox by demonstrating that successful invaders in oasis agroecosystems possess distinctive functional traits that enable them to exploit resource heterogeneity. The oases we studied exemplify this phenomenon, where sharp resource gradients between irrigated agricultural land and surrounding desert are leveraged by invaders with appropriate trait adaptations. With increased human activities, transportation networks and land development enhance propagule dispersal pathways and increase contact with resource-rich microhabitats, raising invasion risk (Eschtruth and Battles, 2009; Milton and Dean, 2010; Rauschert et al., 2017). Our findings revealed that this invasion process is functionally determined—specifically, alien plants with high specific leaf area (SLA) and larger seed mass demonstrated significantly greater invasion potential.

Our study identifies two complementary functional traits driving invasion success in the unique context of oasis agriculture. The significant positive relationship between SLA and invasion risk demonstrates that rapid resource acquisition provides a pivotal advantage in these environments. Plants with high SLA possess thin, broad leaves that enhance photosynthetic efficiency (Liu et al., 2023), allowing them to capitalize on periodic moisture and nutrient pulses from irrigation while maintaining competitive growth rates. Simultaneously, the positive correlation between seed mass and invasion risk represents an adaptation to temporal resource fluctuations. Larger seeds contain more nutrient reserves, improving germination and early seedling survival under stressful conditions (Mechergui et al., 2021). In oasis contexts, where soil moisture fluctuates dramatically between irrigation events, this trait combination—acquisitive leaf strategies (high SLA) for rapid resource capture during favorable periods and larger seeds for sustaining establishment during resource gaps—creates a particularly effective invasion strategy. This trait-based advantage explains why species like *Xanthium strumarium* and *Amaranthus retroflexus* have become problematic in the region’s croplands, where irrigation creates precisely the resource pulses that these trait-equipped strategists can exploit (Larios et al., 2014; Pierce et al., 2017; Wellstein et al., 2017).

The integration of functional traits into species distribution modeling represents a significant contribution of our work. By incorporating SLA as a weighting factor for invasion risk, we achieved exceptionally precise distribution predictions (AUC value of 0.981). This methodological innovation addresses limitations in traditional correlative models by capturing the functional mechanisms underlying successful invasions (Holloway et al., 2016). Our approach recognizes that environmental responses are mediated through traits, with SLA serving as an indicator of species’ responsiveness to environmental gradients (Gong and Gao, 2019; Liu et al., 2021). These insights directly inform management strategies: early detection systems should prioritize species with high SLA and large seed mass, while monitoring efforts should focus on areas supporting high SLA expression. Agricultural practices can be modified through adjusted irrigation regimes, cover cropping, and strategic tillage timing (Damour et al., 2016; Santini et al., 2017). This framework shifts management focus from taxonomic identity to functional mechanisms, offering a more predictive approach for protecting oasis agroecosystems from invasion threats (Funk et al., 2016).

## 5 Conclusion

By analyzing functional traits of 62 alien plant species in oasis agroecosystems, we identified that high specific leaf area (SLA) and larger seed mass are key traits significantly enhancing invasion risk. Given SLA’s high sensitivity to environmental gradient changes, we used it as a weight in MaxEnt models to accurately predict potential distribution areas of high-risk species (AUC = 0.981). These results directly pinpoint the functional traits driving alien plant invasions in oasis agriculture. This study provides a robust foundation for predicting potential invasive species and offers critical insights for developing targeted management strategies to mitigate their impacts on agricultural productivity and ecosystem services in arid regions.

## Data Availability

The original contributions presented in the study are included in the article/**Supplementary Material**. Further inquiries can be directed to the corresponding authors.
