# Supplementary material for: Functional traits driving invasion risk and potential distribution of alien plants in oasis agroecosystems
Source: Front Plant Sci. 2025 May 19;16:1590709. doi: 10.3389/fpls.2025.1590709 (PMC12127345; doi:10.3389/fpls.2025.1590709)
Supplement: Supplementary file 1 [file Table1.docx]

Appendix Table 1. List of All Invasive Species in the Study Area Ranked by Importance Value from Highest to Lowest.

| **No.** | **Species Name** | **Relative Density** | **Relative Frequency** | **Relative Coverage** | **Importance Value** |
| --- | --- | --- | --- | --- | --- |
| 1 | *Amaranthus retroflexus* | 11.952 | 80.360 | 41.723 | 44.678 |
| 2 | *Xanthium strumarium subsp. italicum* | 4.828 | 34.206 | 19.402 | 19.479 |
| 3 | *Abutilon theophrasti Medikus* | 2.791 | 23.622 | 22.929 | 16.447 |
| 4 | *Medicago sativa* | 6.131 | 26.678 | 3.052 | 11.954 |
| 5 | *Cannabis sativa* | 3.572 | 19.149 | 11.513 | 11.411 |
| 6 | *Lactuca serriola* | 6.026 | 14.675 | 7.577 | 9.426 |
| 7 | *Melilotus officinalis* | 1.254 | 8.358 | 13.044 | 7.552 |
| 8 | *Avena fatua* | 1.828 | 8.391 | 1.910 | 4.043 |
| 9 | *Cuscuta campestris* | 0.501 | 6.929 | 1.363 | 2.931 |
| 10 | *Melilotus albus* | 0.846 | 4.965 | 2.703 | 2.838 |
| 11 | *Xanthium spinosum* | 1.416 | 4.583 | 2.403 | 2.801 |
| 12 | *Ambrosia artemisiifolia* | 1.702 | 1.473 | 4.084 | 2.420 |
| 13 | *Atriplex canescens* | 1.135 | 4.103 | 1.934 | 2.391 |
| 14 | *Ambrosia trifida* | 1.671 | 1.473 | 4.002 | 2.382 |
| 15 | *Datura stramonium* | 0.755 | 4.495 | 1.752 | 2.334 |
| 16 | *Lolium perenne* | 0.089 | 3.072 | 2.435 | 1.865 |
| 17 | *Amaranthus albus* | 0.381 | 2.990 | 1.524 | 1.632 |
| 18 | *Daucus carota* | 0.119 | 1.091 | 3.378 | 1.529 |
| 19 | *Trifolium pratense* | 0.851 | 2.073 | 1.489 | 1.471 |
| 20 | *Amaranthus blitum* | 0.148 | 3.077 | 1.183 | 1.469 |
| 21 | *Chenopodiastrum hybridum* | 0.688 | 1.637 | 1.709 | 1.345 |
| 22 | *Lolium multiflorum* | 0.300 | 1.833 | 1.322 | 1.152 |
| 23 | *Amaranthus blitoides* | 0.680 | 2.397 | 0.365 | 1.147 |
| 24 | *Ipomoea purpurea* | 0.155 | 2.455 | 0.716 | 1.109 |
| 25 | *Helianthus tuberosus* | 0.368 | 1.637 | 1.074 | 1.026 |
| 26 | *Hibiscus trionum* | 0.094 | 0.709 | 2.151 | 0.985 |
| 27 | *Geranium carolinianum* | 0.715 | 1.309 | 0.578 | 0.867 |
| 28 | *Cosmos bipinnatus* | 0.431 | 1.473 | 0.690 | 0.865 |
| 29 | *Erigeron canadensis* | 0.153 | 0.491 | 1.683 | 0.776 |
| 30 | *Zinnia peruviana* | 0.071 | 1.200 | 0.817 | 0.696 |
| 31 | *Mirabilis jalapa* | 0.017 | 0.436 | 0.932 | 0.462 |
| 32 | *Centaurea cyanus* | 0.020 | 0.466 | 0.867 | 0.451 |
| 33 | *Parthenocissus quinquefolia* | 0.024 | 0.436 | 0.798 | 0.419 |
| 34 | *Tagetes erecta* | 0.022 | 0.655 | 0.471 | 0.383 |
| 35 | *Cenchrus echinatus* | 0.036 | 0.546 | 0.504 | 0.362 |
| 36 | *Bromus catharticus* | 0.004 | 0.164 | 0.697 | 0.288 |
| 37 | *Erigeron annuus* | 0.047 | 0.655 | 0.163 | 0.288 |
| 38 | *Xanthium chinense* | 0.011 | 0.480 | 0.326 | 0.272 |
| 39 | *Erigeron bonariensis* | 0.011 | 0.600 | 0.089 | 0.233 |
| 40 | *Ricinus communis* | 0.006 | 0.218 | 0.452 | 0.225 |
| 41 | *Sonchus asper* | 0.096 | 0.491 | 0.089 | 0.225 |
| 42 | *Amaranthus hybridus* | 0.010 | 0.436 | 0.225 | 0.224 |
| 43 | *Amaranthus viridis* | 0.019 | 0.327 | 0.312 | 0.219 |
| 44 | *Oenothera biennis* | 0.006 | 0.327 | 0.274 | 0.202 |
| 45 | *Amaranthus cruentus* | 0.008 | 0.281 | 0.192 | 0.160 |
| 46 | *Dysphania ambrosioides* | 0.007 | 0.251 | 0.119 | 0.126 |
| 47 | *Senecio vulgaris* | 0.006 | 0.120 | 0.148 | 0.091 |
| 48 | *Veronica persica* | 0.002 | 0.109 | 0.148 | 0.086 |
| 49 | *Cuscuta epilinum* | 0.006 | 0.142 | 0.104 | 0.084 |
| 50 | *Lolium remotum* | 0.006 | 0.109 | 0.128 | 0.081 |
| 51 | *Bidens frondosa* | 0.002 | 0.055 | 0.184 | 0.080 |
| 52 | *Phytolacca americana* | 0.002 | 0.109 | 0.119 | 0.077 |
| 53 | *Gypsophila vaccaria* | 0.002 | 0.109 | 0.116 | 0.076 |
| 54 | *Datura innoxia* | 0.004 | 0.133 | 0.089 | 0.075 |
| 55 | *Galinsoga parviflora* | 0.009 | 0.164 | 0.014 | 0.062 |
| 56 | *Galinsoga quadriradiata* | 0.005 | 0.164 | 0.019 | 0.063 |
| 57 | *Coreopsis lanceolata* | 0.005 | 0.109 | 0.060 | 0.058 |
| 58 | *Gaillardia pulchella* | 0.003 | 0.065 | 0.089 | 0.052 |
| 59 | *Lepidium virginicum* | 0.001 | 0.022 | 0.116 | 0.046 |
| 60 | *Ipomoea hederacea* | 0.001 | 0.055 | 0.079 | 0.045 |
| 61 | *Amorpha fruticosa* | 0.001 | 0.055 | 0.025 | 0.027 |
| 62 | *Euphorbia marginata* | 0.001 | 0.055 | 0.017 | 0.024 |
