# Supplementary material for: Functional traits driving invasion risk and potential distribution of alien plants in oasis agroecosystems
Source: Front Plant Sci. 2025 May 19;16:1590709. doi: 10.3389/fpls.2025.1590709 (PMC12127345; doi:10.3389/fpls.2025.1590709)
Supplement: Supplementary file 2 [file Table2.docx]

Appendix Table 2. Statistical Table of Growth Traits, Reproductive Traits, and Dispersal Traits of Alien Plants.

| **Species Name** | **Invasion**  **Risk** | **Life**  **Forms** | **Average**  **SLA**  **（cm^2^/g）** | **Average Plant Height（cm）** | **Mating**  **System** | **Flowering**  **Duration（month）** | **Pollination**  **Method** | **Average**  **Grain Weight （g）** | **Fruit**  **Type** | **Diffusion**  **Mode** |
| --- | --- | --- | --- | --- | --- | --- | --- | --- | --- | --- |
| *Amaranthus retroflexus* | high | Ah | 362 | 90.5 | Selfing | 2 | Anemophily | 0.272 | Utricle | Gravity |
| *Xanthium strumarium subsp. italicum* | high | Ah | 335 | 181.6 | Outcrossing | 2 | Anemophily | 13.077 | Achene | Animal |
| *Abutilon theophrasti Medikus* | high | Ah | 289 | 153.6 | Outcrossing | 3 | Entomophily | 1.612 | Capsule | Machinery |
| *Medicago sativa* | high | Ph | 402 | 77.1 | Outcrossing | 3 | Entomophily | 1.597 | Legume | Gravity |
| *Cannabis sativa* | high | Ah | 219 | 190.4 | Outcrossing | 2 | Anemophily | 3.924 | Achene | Gravity |
| *Lactuca serriola* | high | ABh | 426 | 71.6 | Selfing | 2 | Anemophily | 0.278 | Achene | Wind |
| *Melilotus officinalis* | high | ABh | 347 | 128.7 | Outcrossing | 3 | Entomophily | 1.599 | Legume | Gravity |
| *Avena fatua* | high | Ah | 246 | 117.3 | Selfing | 3 | Anemophily | 3.073 | Caryopsis | Gravity |
| *Cuscuta campestris* | high | Ah | 302 | 15.8 | Selfing | 3 | Anemophily | 0.116 | Capsule | Animal |
| *Melilotus albus* | high | ABh | 238 | 141.5 | Outcrossing | 3 | Entomophily | 1.435 | Legume | Gravity |
| *Xanthium spinosum* | high | Ah | 224 | 79.8 | Outcrossing | 2 | Anemophily | 14.773 | Achene | Machinery |
| *Ambrosia artemisiifolia* | high | Ah | 317 | 130.4 | Outcrossing | 2 | Anemophily | 0.592 | Achene | Gravity |
| *Atriplex canescens* | high | Ps | 118 | 153.8 | Outcrossing | 2 | Anemophily | 0.776 | Utricle | Wind |
| *Ambrosia trifida* | high | Ah | 285 | 249.3 | Outcrossing | 2 | Anemophily | 2.161 | Multiple | Gravity |
| *Datura stramonium* | high | Ah | 421 | 87.9 | Outcrossing | 5 | Entomophily | 1.711 | Capsule | Gravity |
| *Lolium perenne* | medium | Ph | 185 | 81.4 | Outcrossing | 3 | Anemophily | 1.205 | Caryopsis | Gravity |
| *Amaranthus albus* | medium | Ah | 238 | 64.2 | Outcrossing | 2 | Anemophily | 0.269 | Utricle | Wind |
| *Daucus carota* | medium | Bh | 602 | 102.8 | Outcrossing | 3 | Entomophily | 0.736 | Dehiscent | Wind |
| *Trifolium pratense* | medium | Ph | 210 | 28.3 | Outcrossing | 5 | Entomophily | 0.339 | Legume | Gravity |
| *Amaranthus blitum* | medium | Ah | 237 | 35.4 | Outcrossing | 3 | Anemophily | 0.136 | Utricle | Wind |
| *Chenopodiastrum hybridum* | medium | Ah | 300 | 102.8 | Selfing | 2 | Anemophily | 0.264 | Utricle | Gravity |
| *Lolium multiflorum* | medium | Ah | 225 | 96.3 | Outcrossing | 2 | Anemophily | 1.179 | Caryopsis | Gravity |
| *Amaranthus blitoides* | medium | Ah | 275 | 41.6 | Outcrossing | 2 | Anemophily | 0.209 | Utricle | Gravity |
| *Ipomoea purpurea* | medium | Ah | 168 | 27.3 | Outcrossing | 6 | Entomophily | 4.026 | Capsule | Gravity |
| *Helianthus tuberosus* | medium | Ph | 233 | 110.5 | Outcrossing | 2 | Entomophily | 3.116 | Achene | Wind |
| *Hibiscus trionum* | medium | Ah | 253 | 27.1 | Outcrossing | 3 | Entomophily | 1.162 | Capsule | Gravity |
| *Geranium carolinianum* | medium | Ph | 325 | 29.5 | Outcrossing | 4 | Entomophily | 0.291 | Capsule | Gravity |
| *Cosmos bipinnatus* | medium | APh | 225 | 130.5 | Outcrossing | 3 | Entomophily | 0.836 | Achene | Wind |
| *Erigeron canadensis* | medium | Ah | 175 | 67.2 | Selfing | 5 | Anemophily | 0.026 | Achene | Wind |
| *Zinnia peruviana* | medium | Ah | 225 | 20.1 | Outcrossing | 5 | Entomophily | 1.305 | Achene | Gravity |
| *Mirabilis jalapa* | low | Ah | 305 | 90.5 | Outcrossing | 5 | Entomophily | 11.672 | Achene | Gravity |
| *Centaurea cyanus* | low | ABh | 172 | 52.4 | Outcrossing | 4 | Entomophily | 3.194 | Achene | Gravity |
| *Parthenocissus quinquefolia* | low | Wv | 288 | 12.4 | Outcrossing | 2 | Entomophily | 5.216 | Achene | Animal |
| *Tagetes erecta* | low | Ah | 159 | 31.5 | Outcrossing | 3 | Entomophily | 0.603 | Achene | Gravity |
| *Cenchrus echinatus* | low | Ah | 197 | 41.8 | Selfing | 3 | Anemophily | 1.273 | Caryopsis | Animal |
| *Bromus catharticus* | low | Ah | 187 | 102.3 | Selfing | 2 | Anemophily | 2.372 | Caryopsis | Gravity |
| *Erigeron annuus* | low | ABh | 277 | 97.5 | Outcrossing | 3 | Entomophily | 0.058 | Achene | Wind |
| *Xanthium chinense* | low | Ah | 165 | 89.2 | Outcrossing | 3 | Anemophily | 7.916 | Achene | Machinery |
| *Erigeron bonariensis* | low | ABh | 133 | 63.7 | Outcrossing | 3 | Anemophily | 0.043 | Achene | Wind |
| *Ricinus communis* | low | Ah | 177 | 235.9 | Outcrossing | 3 | Anemophily | 18.103 | Capsule | Gravity |
| *Sonchus asper* | low | Ah | 182 | 37.9 | Selfing | 4 | Entomophily | 0.089 | Achene | Wind |
| *Amaranthus hybridus* | low | Ah | 182 | 39.3 | Outcrossing | 2 | Anemophily | 0.253 | Utricle | Gravity |
| *Amaranthus viridis* | low | Ah | 236 | 69.3 | Selfing | 3 | Anemophily | 0.168 | Utricle | Gravity |
| *Oenothera biennis* | low | Bh | 203 | 79.2 | Outcrossing | 3 | Entomophily | 0.897 | Capsule | Wind |
| *Amaranthus cruentus* | low | Ah | 81 | 149.6 | Outcrossing | 2 | Anemophily | 0.405 | Utricle | Gravity |
| *Dysphania ambrosioides* | low | APh | 265 | 60.5 | Selfing | 2 | Anemophily | 0.091 | Utricle | Gravity |
| *Senecio vulgaris* | no | Ah | 155 | 23.9 | Selfing | 7 | Anemophily | 0.145 | Achene | Wind |
| *Veronica persica* | no | ABh | 206 | 32.1 | Outcrossing | 3 | Entomophily | 0.011 | Capsule | Wind |
| *Cuscuta epilinum* | no | Ah | 207 | 11.3 | Outcrossing | 3 | Entomophily | 0.118 | Capsule | Animal |
| *Lolium remotum* | no | Ah | 129 | 66.3 | Outcrossing | 2 | Anemophily | 0.261 | Caryopsis | Gravity |
| *Bidens frondosa* | no | Ah | 211 | 66.5 | Outcrossing | 2 | Entomophily | 1.308 | Achene | Machinery |
| *Phytolacca americana* | no | Ph | 212 | 167.3 | Outcrossing | 3 | Entomophily | 1.098 | Bacca | Animal |
| *Gypsophila vaccaria* | no | ABh | 175 | 50.1 | Outcrossing | 3 | Entomophily | 0.866 | Capsule | Gravity |
| *Datura innoxia* | no | Ah | 299 | 113.5 | Outcrossing | 3 | Entomophily | 1.087 | Capsule | Gravity |
| *Galinsoga quadriradiata* | no | Ah | 252 | 22.1 | Selfing | 3 | Entomophily | 0.132 | Achene | Wind |
| *Galinsoga parviflora* | no | Ah | 268 | 20.7 | Selfing | 3 | Entomophily | 0.108 | Achene | Wind |
| *Coreopsis lanceolata* | no | Ph | 124 | 59.1 | Outcrossing | 5 | Entomophily | 0.317 | Achene | Wind |
| *Gaillardia pulchella* | no | Ah | 156 | 29.1 | Outcrossing | 3 | Entomophily | 0.942 | Achene | Wind |
| *Lepidium virginicum* | no | ABh | 112 | 68.8 | Selfing | 2 | Anemophily | 0.097 | Silicle | Machinery |
| *Ipomoea hederacea* | no | Ah | 167 | 18.1 | Outcrossing | 3 | Entomophily | 5.208 | Capsule | Gravity |
| *Amorpha fruticosa* | no | Ds | 333 | 233.5 | Outcrossing | 4 | Entomophily | 3.343 | Legume | Animal |
| *Euphorbia marginata* | no | Ah | 251 | 70.5 | Outcrossing | 3 | Entomophily | 0.576 | Capsule | Gravity |

Note: In the abbreviations of life forms, Ph stands for perennial herb, Ps for perennial shrub, Bh for biennial herb, Ds for deciduous shrub, Wv for woody vine, Ah for annual herb, APh for annual or perennial herb, and ABh for annual or biennial herb.
